# Phosphate-deprivation and damage signalling by extracellular ATP

**DOI:** 10.3389/fpls.2022.1098146

**Published:** 2023-01-12

**Authors:** Elsa Matthus, Youzheng Ning, Fahad Shafiq, Julia M. Davies

**Affiliations:** ^1^ Department of Plant Sciences, University of Cambridge, Cambridge, United Kingdom; ^2^ Leibniz Centre for Agricultural Landscape Research (ZALF), Müncheberg, Germany; ^3^ Institute of Molecular Biology and Biotechnology (IMBB), The University of Lahore, Punjab, Pakistan

**Keywords:** phosphate deprivation, ATP and damage signalling, calcium, DAMP, immunity, phosphate, root

## Abstract

Phosphate deprivation compromises plant productivity and modulates immunity. DAMP signalling by extracellular ATP (eATP) could be compromised under phosphate deprivation by the lowered production of cytosolic ATP and the need to salvage eATP as a nutritional phosphate source. Phosphate-starved roots of *Arabidopsis* can still sense eATP, indicating robustness in receptor function. However, the resultant cytosolic free Ca^2+^ signature is impaired, indicating modulation of downstream components. This perspective on DAMP signalling by extracellular ATP (eATP) addresses the salvage of eATP under phosphate deprivation and its promotion of immunity, how Ca^2+^ signals are generated and how the Ca^2+^ signalling pathway could be overcome to allow beneficial fungal root colonization to fulfill phosphate demands. Safe passage for an endophytic fungus allowing root colonization could be achieved by its down-regulation of the Ca^2+^ channels that act downstream of the eATP receptors and by also preventing ROS accumulation, thus further impairing DAMP signalling.

## Introduction

Phosphate (Pi) deprivation is readily experienced in the field without fertilizer input (Alewell et al., 2020) and leads to lower cellular and cytosolic Pi levels within minutes (Duff et al., 1989; Pratt et al., 2009). Deficiency triggers a shift to alternative metabolic pathways which consume less Pi (Duff et al., 1989; Plaxton and Tran, 2011; Pant et al., 2015) and phosphorylated metabolites decrease (Pant et al., 2015). Phospholipids are remodelled into sulpho- and glycolipids, restricted to the cytoplasmic leaflet of the plasma membrane (Andersson et al., 2005; Tjellström et al., 2010; Nakamura, 2013; Okazaki et al., 2013). This remodelling could be part of the restructuring of signalling systems. Indeed, Pi deprivation attenuates the cytosolic free Ca^2+^ ([Ca^2+^]_cyt_) signalling response to mechanical stress, salinity, and osmotic stress in *Arabidopsis* roots (Matthus et al., 2019a; Matthus et al., 2022).

It is now increasingly recognized that Pi availability and homeostasis are intricately linked with plant immunity signalling (Castrillo et al., 2017; Dindas et al., 2022; Tang et al., 2022; Val-Torregrosa et al., 2022). Under low Pi, plants initiate the Phosphate Starvation Response (PSR) driven by the MYB transcription factor Phosphate Starvation Response1 (PHR1) to modulate not only growth and metabolism but the composition of the plant’s microbiota (well beyond the interaction with mycorrhizal fungi) to favour those mineralizing poorly accessible Pi sources to promote Pi nutrition. Achieving this may involve modulating immunity, indeed PHR1 negatively regulates transcription of genes responding to the Pathogen Associated Molecular Pattern (PAMP) bacterial peptide flg22 (Castrillo et al., 2017; Isidra-Arellano et al., 2021). In roots of *Arabidopsis thaliana* (as a non-host for mycorrhizal fungi), part of the PSR is the production of a subset of anti-immunity RALF (Rapid Alkalinization Factor) peptides that are perceived by the plasma membrane Feronia receptor. This is then thought to disrupt perception of flg22 by the FLS2/BAK1 (BRASSINOSTEROID INSENSITIVE 1-ASSOCIATED RECEPTOR KINASE 1) receptor complex to lower immunity (Tang et al., 2022). However in Pi-starved *Arabidopsis* root hairs the abundance of the high affinity PHT1.4 transporter is increased in the PSR but PAMPs (including flg22) were found to act through BIK1 to inhibit PHT1.4-mediated Pi uptake (Dindas et al., 2022). Nevertheless, Pi-starved mutants lacking PHT1.4 were less susceptible than wild type to infection by a bacterial pathogen (*Ralstonia solanacearum*), placing this component as a negative regulator of immunity and consistent with PSR’s modulating defence (Dindas et al., 2022).

Wounding and the presence of microbes causes accumulation of extracellular ATP (eATP) by plants. Mechanical wounding of *Arabidopsis* roots (Weerasinghe et al., 2009; Dark et al., 2011) and leaves (Song et al., 2006; Myers Jr et al., 2022) increases eATP, consistent with breaches of the plasma membrane’s permitting efflux of cytosolic ATP. The effect is not limited to *Arabidopsis*; wounding cells of the macroalga *Dasycladus vermicularis*, roots of carrot (*Daucus carota*; Gastélum-Estrada et al., 2020) and leaves of kidney bean (*Phaseolus vulgaris L*) also causes eATP accumulation (Wang et al., 2019). eATP accumulation by *Arabidopsis* leaves can occur in response to flg22 and *Pseudomonas syringae* (Chen et al., 2017) but in those cases the mechanistic basis of eATP accumulation is unknown. For roots, eATP increases in barley (*Hordeum vulgare*) and *Arabidopsis* during colonization by the fungal endophyte *Serendipita indica* (Nizam et al., 2019). The significance of such eATP accumulation lies in eATP’s ability to signal wounding or microbial presence as a constitutive DAMP (Damage Associated Molecular Pattern; Choi et al., 2014; Tanaka and Heil, 2021). A constitutive DAMP is a molecule that is present before damage and becomes a signal on moving passively from its “normal” site as a consequence of damage (Tanaka and Heil, 2021); for ATP, this is moving from the cytosol to the extracellular face of the plasma membrane. eATP’s acting as a DAMP is conserved across kingdoms, working in animals and fungi as well as plants but signalling systems differ markedly (Medina-Castellanos et al., 2014; Medina-Castellanos et al., 2018; Verkhratsky, 2021). Studies on eATP signalling are usually conducted on plants grown under optimal nutrient conditions. Given the apparent need to conserve Pi, utilising a Pi-rich signalling molecule such as eATP potentially places plants suffering from Pi deprivation at risk of impaired signalling outcomes. However, in light of immunity modulation in the PSR, this could be a necessary and beneficial risk. After outlining the eATP signalling pathway in defence, this Perspective considers how Pi deprivation is currently known to affect it, how eATP as a nutritional Pi source may link with defence and argues that (although modulated) eATP signalling will remain a key line of defence for microbes to overcome under this abiotic stress.

## eATP signalling intersects with multiple pathways

The eATP-regulated *Arabidopsis* transcriptome is enriched in immune- and wound-response genes (Jeter et al., 2004; Choi et al., 2014; Tripathi et al., 2018; Jewell et al., 2019; Jewell et al., 2022). The signalling pathway from eATP to wounding/immunity transcription runs through the plasma membrane legume-like lectin serine-threonine receptor kinase “DOes not Respond to Nucleotides1” (DORN1/P2K1) and also its co-receptor phosphorylation target P2K2, although whether all cell types deploy this co-receptor is unknown (Choi et al., 2014; Jewell et al., 2019; Pham et al., 2020). Wound-induced inhibition of plant growth is mediated by P2K1 (Shi et al., 2022) and this receptor is required for limiting infection by bacteria, oomycetes and fungi (Gouget et al., 2006; Bouwmeester et al., 2011; Bouwmeester et al., 2014; Balagué et al., 2017; Chen et al., 2017; Tripathi et al., 2018; Nizam et al., 2019; Kumar et al., 2020). Overexpression of *P2K1* can confer resistance to insect and nematode attack (Jewell et al., 2022). Potential eATP receptors as P2K1 orthologues have been reported in *Camelina sativa* (Li et al., 2016) and banana (*Musa acuminata*; Shan et al., 2020) for example, but there are no reports on cereals. These contain large families of legume-like lectin serine-threonine receptor kinase genes for testing (72 in *Oryza sativa* (rice) and 84 in *Triticum aestivum* (bread wheat): Vaid et al., 2012; Shumayla et al., 2016).

**Figure 1:**
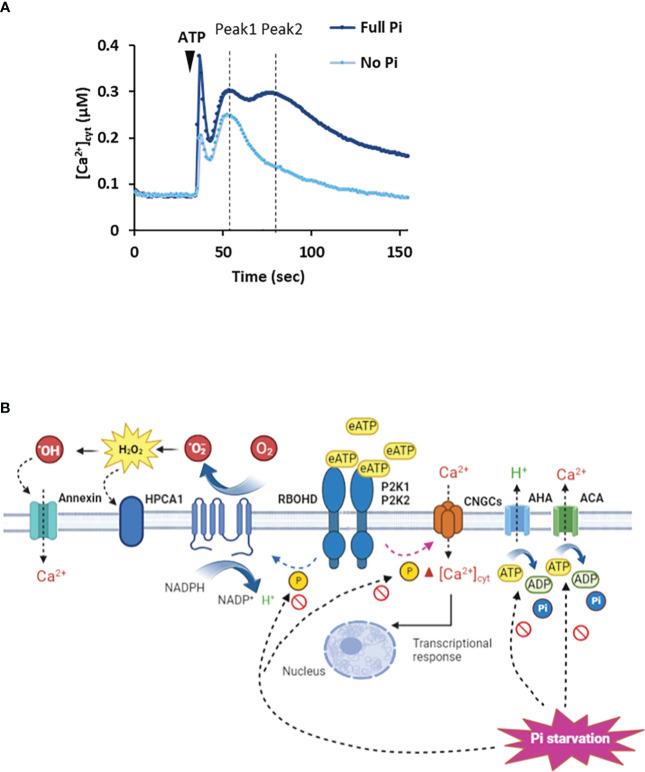
Extracellular ATP causes elevation of [Ca^2+^]_cyt_. **(A)** *Arabidopsis* roots or root tips (expressing cytosolic aequorin as a [Ca^2+^]_cyt_ reporter) grown in full Pi medium respond to ATP addition with an initial [Ca^2+^]_cyt_ increase caused by mechanical perturbation, followed by an ATP-induced biphasic increase (Peak 1, Peak 2). In Pi-starved roots, the magnitude of the Peak responses is lessened. Schema based on results of Matthus et al., 2019a; Matthus et al., 2019b; Mohammad-Sidik et al., 2021; Matthus et al., 2022; Wang et al., 2022a. **(B)** In Pi-replete *Arabidopsis*, extracellular ATP (eATP) is recognized by the plasma membrane P2K1 and P2K2 receptors. This can lead to opening of CNGC channels by an unknown mechanism to elevate [Ca^2+^]_cyt_ (Sun et al., 2021; Wu et al., 2021; Duong et al., 2022; Wang et al., 2022a). In guard cells, P2K1 can activate RBOHD by phosphorylation (Chen et al., 2017) whilst in roots its target may be RBOHC (Demidchik et al., 2009). The resultant extracellular ROS could be sensed by the HPCA1 hydrogen peroxide receptor (Wu et al., 2020) although there is no evidence for this yet. Peroxide could enter the cytosol through aquaporins (not shown) or be converted to hydroxyl radicals to activate Annexin1. H^+^-ATPases (AHA) promote hyperpolarised membrane voltage (Haruta and Sussman, 2012) to facilitate Ca^2+^ channel opening whilst Ca^2+^-ATPase activity would help terminate the [Ca^2+^]_cyt_ signal (Costa et al., 2017). Under Pi deprivation, cytosolic ATP limitation may impair AHA/Ca^2+^-ATPase activity and potentially the phosphorylation activity of the receptors. The involvement of P2K2 may be questioned (Matthus et al., 2022). The identities of the Ca^2+^ channels may change and the involvement of RBOHs has yet to be determined.

In *Arabidopsis* Pi-replete roots, eATP causes a biphasic increase in [Ca^2+^]_cyt_ as a second messenger with the first phase generated by the apex followed by a second, sub-apical phase (**Figure 1A**: Matthus et al., 2019a; Matthus et al., 2019b; Mohammad-Sidik et al., 2021; Matthus et al., 2022; Wang et al., 2022a). This response appears to have an absolute requirement for P2K1 but recently Matthus et al. (2022) reported a small but significant eATP-induced [Ca^2+^]_cyt_ increase in roots that was independent of this receptor. In *Arabidopsis* root epidermis (Pi-replete), P2K1 and P2K2 cause an initial Ca^2+^ influx mediated by the plasma membrane Cyclic Nucleotide Gated Channel CNGC2 (**Figure 1B**: Wang et al., 2022a). Another CNGC, CNGC6, may also be involved in the root’s response (Duong et al., 2022). CNGC2 is also part of the eATP pathway in cotyledons and pollen grain (Sun et al., 2021; Wu et al., 2021), although its involvement in roots appears restricted to the epidermis (Wang et al., 2022a). CNGC2 may form a connection with PAMP-triggered immunity as it can also operate in flg22 signalling (Tian et al., 2019) and it also works in Jasmonic Acid signalling (Lu et al., 2016). CNGC2 may also form an intersect with abiotic stress signalling and development as it is involved in heat stress signalling (Finka et al., 2012; Katano et al., 2018), high light signalling (Fichman et al., 2021), response to auxin (Chakraborty et al., 2021) and floral transition (Chin et al., 2013). How the P2K1 and P2K2 eATP receptors promote opening of CNGC2 remains unknown; possibilities include phosphorylation or production of cyclic mononucleotides by cryptic cyclase domains (Sun et al., 2021). The resultant elevation of [Ca^2+^]_cyt_ by CNGC2 or other Ca^2+^ channels (Wilkins et al., 2016; Jarratt-Barnham et al., 2021) may link to the transcriptional response through downstream elevation of nuclear Ca^2+^ (Krebs et al., 2012; Loro et al., 2012) and breakdown of the Calmodulin-binding Transcription Activator3, CAMTA3 (Jewell et al., 2019; Jiang et al., 2020).

## Pi deprivation influences the eATP-induced [Ca^2+^]_cyt_ signatures

*Arabidopsis* roots deprived of Pi can still respond to eATP with a distinct [Ca^2+^]_cyt_ increase or “signature” (Matthus et al., 2019a; Matthus et al., 2022). There is no substitution of P2K1 in Pi-deprived roots, it is still absolutely required for the [Ca^2+^]_cyt_ response (Matthus et al., 2022), indicating a robust perception system that withstands perturbation. Indeed, expression of *P2K1* does not respond significantly to Pi starvation whilst P2K1 abundance in roots can even increase (Lin et al., 2011; Lan et al., 2012; Zhang et al., 2020). However, the P2K1-independent component of the [Ca^2+^]_cyt_ signature was lost on Pi deprivation (Matthus et al., 2022). It could be that this component was generated by P2K2 or unknown receptors, for which evidence is accumulating (Zhu et al., 2017; Matthus et al., 2019b; Zhu R. et al., 2020; Pham et al., 2020; Smith et al., 2021). Although Pi-deprived *Arabidopsis* roots can still respond to eATP, the spatio-temporal pattern of the [Ca^2+^]_cyt_ increase is altered with a significantly lower first phase and the abolition of the second, sub-apical response (**Figure 1A**: Matthus et al., 2019a; Matthus et al., 2022). The downstream consequences of this change are unknown. The position where the sub-apical [Ca^2+^]_cyt_ increase should occur corresponded with a region of increased cytosolic Reactive Oxygen Species (ROS), most likely hydrogen peroxide (Matthus et al., 2019a). This effect on the [Ca^2+^]_cyt_ signal increased over days of Pi starvation and was linked to Fe availability (a normal response was restored by Fe deprivation; Matthus et al., 2019a). In plant signalling systems, ROS are held to amplify or propagate [Ca^2+^]_cyt_ increase by modulating Ca^2+^ transporters (Demidchik and Shabala, 2018). In contrast, under Pi deprivation ROS ostensibly limits the [Ca^2+^]_cyt_ response to eATP. Whether the impaired [Ca^2+^]_cyt_ signal is the result of different complements of Ca^2+^ transporters (Shukla et al., 2021) as a consequence of Pi deprivation (possibly affecting the links with other pathways) and/or different regulatory mechanisms now needs to be determined. For the latter, it may be relevant that lowered cytosolic ATP (see section below) affects actin dynamics (Dai et al., 2022; Wang et al., 2022b) that could regulate plasma membrane Ca^2+^ channels (Qian and Xiang, 2019). It may also be relevant that lower cytosolic ATP could impair the activity of plasma membrane H^+^-ATPases, possibly impairing activity of voltage-dependent plasma membrane Ca^2+^ channels. This could explain why the *Arabidopsis* mutant lacking a major H^+^-ATPase isoform (AHA2) has a lower [Ca^2+^]_cyt_ response to eATP (Haruta and Sussman, 2012).

## eATP signalling under Pi deprivation – malnourished defence

Cellular ATP level drops sharply in response to Pi deprivation as shown in kidney bean roots, *Catharanthus roseus* and sycamore cell culture (Gniazdowska et al., 1998; Shimano and Ashihara, 2006; Gout et al., 2014). Although gradients of cytosolic Mg-ATP can be resolved at cellular level with imaging of the ATeam 1.03-nD/nA reporter (De Col et al., 2017), the effect of Pi deprivation remains untested. The drop in cellular ATP begs the questions of whether Pi-deprived tissues continue to maintain their basal eATP levels (with the possibility of too low a level triggering cell death; Chivasa et al., 2005) and whether wound/pathogen-induced eATP increases would be significantly lower. For Pi-replete *Arabidopsis*, wound-induced eATP estimates range from 35 nM to 45 µM (Song et al., 2006; Dark et al., 2011; Myers Jr et al., 2022). There appear to be no reports on the effect of Pi-deprivation in the literature although Tawaraya et al. (2014) reported that ADP was no longer present in the root exudates of Pi-deprived soybean roots.

**Figure 2:**
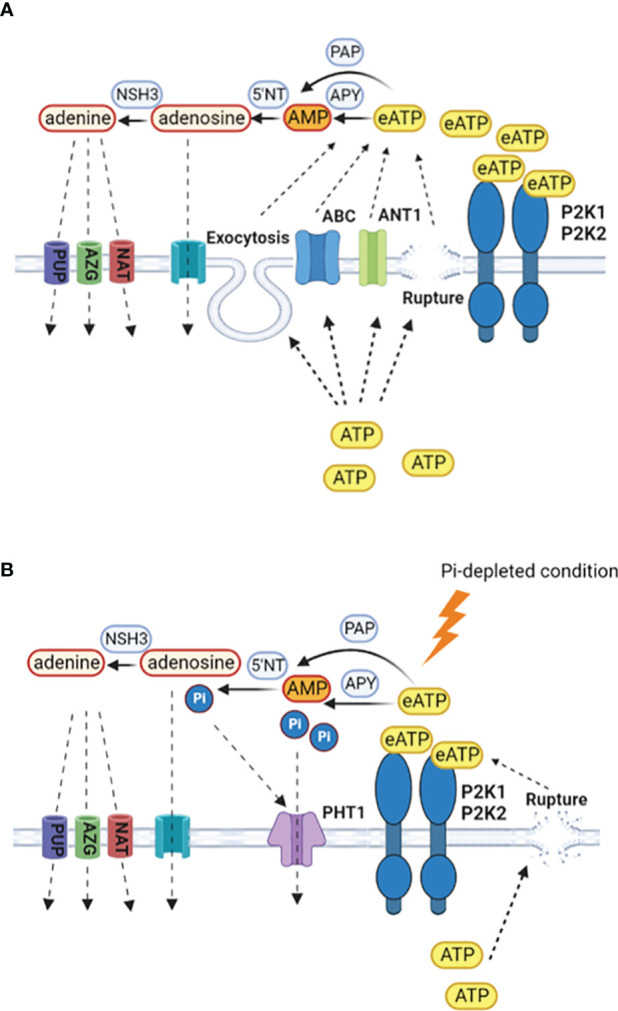
Production and scavenging of eATP. **(A)** In Pi-replete conditions, cytosolic ATP may be released to the extracellular space by wounding, exocytosis or specific transporters such as ANT1 (Thomas et al., 2000; Kim et al., 2006; Rieder and Neuhaus, 2011; Wu et al., 2011). Hydrolysis of eATP to terminate signalling may be by apyrases (APY), purple acid phosphatases (PAP), with subsequent breakdown by 5´nucleotidases and nucleoside hydrolases (Liang et al., 2010; Tran et al., 2010; Tian et al., 2012; Wang et al., 2014; Mehra et al., 2017; Kavka et al., 2021; Zhu S. et al., 2020; Clark et al., 2021). Retrieval of adenosine would be by equilibrative nucleoside transporters and for adenine by purine permeases (PUP), azaguanine resistant proteins (AZG) and nucleobase-ascorbate transporter family members (NAT) (Gillissen et al., 2000; Bernard et al., 2011; Witte and Herde, 2020). **(B)** Under Pi deprivation, there may be less cytosolic ATP to export and export systems could be limited, with wounding being the predominant route. P2K1 still appears competent but the involvement of P2K2 may be questioned (Matthus et al., 2022). Enzymes involved in eATP breakdown would become part of a Pi salvage system, with Pi uptake by PHT1 high affinity transporters that are induced by the PSR. Given the negative role of PHT1.4 in immunity (Dindas et al., 2022), the identities of the PHT1s may be an important control point in determining the resultant balance between nutrition and immunity.

Conservation of cytosolic ATP could involve restricting non-wounding ATP efflux pathways at the plasma membrane that are thought to include ABC transporters and anion channels (Thomas et al., 2000; Rieder and Neuhaus, 2011; Wu et al., 2011; Witte and Herde, 2020: **Figure 2**). Restricted growth caused by Pi deprivation could also limit cytosolic ATP release by exocytosis (Kim et al., 2006). However, as growing root hairs accumulate eATP at their apices (Kim et al., 2006), these levels might still be retained as root hair elongation increases as a potential mechanism to access soil Pi in the absence of mutualistic microbial partners. Indeed, lowering eATP can inhibit root hair elongation (Clark et al., 2010). Moreover, eATP may have a role to play in legume root hair deformation (curling) in response to nodulation factors. Curling is a re-orientation of elongative polar growth towards the nodulation factor (Esseling et al., 2003) and under Pi deprivation, significantly fewer *Phaseolus vulgaris* root hairs can curl, compromising the extent of the rhizobial symbiosis (Isidra-Arellano et al., 2018). The implication is that lowered Pi lowers root hair eATP and re-orientation is compromised. One effect of lowered eATP could be impaired ROS production at the root hair apex, which helps drive polar growth (Foreman et al., 2003; Cárdenas et al., 2008).

Scavenging of eATP so that it can be utilised as a nutritional Pi source is evident in a wide range of plants including beech and poplar trees (Scheerer et al., 2019). Some extracellular Purple Acid Phosphatase (PAP) isoforms can scavenge eATP as a Pi source in *Arabidopsis*, *Phaseolus vulgaris*, poplar, rice and soybean, with production of some isoforms increasing upon Pi deprivation (Liang et al., 2010; Tran et al., 2010; Tian et al., 2012; Wang et al., 2014; Mehra et al., 2017; Kavka et al., 2021; Zhu S. et al., 2020; **Figure 2**). Apyrases hydrolyse ATP and are found in Golgi/ER, plasma membrane and apoplast (Summers et al., 2017; Clark et al., 2021). Apyrases could also scavenge eATP as a Pi source or limit its export, indeed there is an inverse relationship between their expression and eATP concentration around roots (Thomas et al., 1999; Lim et al., 2014; Deng et al., 2015). Studies suggest that regulation of ecto-apyrase may be critical to infection by *Rhizobia* and mycorrhizal fungi, such that their lowering of eATP promotes infection (Kalsi and Etzler, 2000; Govindarajulu et al., 2009; Roberts et al., 2013). It is held that the AMP produced by ecto-apyrase could be converted to adenosine then adenine by 5´nucleotidases and nucleoside hydrolases with uptake of those end products possibly by equilibrative nucleoside transporters (ENT) for adenosine and for adenine by the purine permeases, azaguanine resistant proteins and nucleobase-ascorbate transporter family members (Gillissen et al., 2000; Bernard et al., 2011; Witte and Herde, 2020; **Figure 2**). Efficient salvage of adenosine appears critical given that its accumulation compromises the resistance to *Botrytis cinerea* that is afforded by P2K1 (Daumann et al., 2015; Tripathi et al., 2018). Salvage of adenine should promote cytosolic ATP content (Dai et al., 2022).

As a non-mycorrhizal host, *Arabidopsis* roots allow colonization by fungal endophytes such as *Colletotrichum tofieldiae* and *Serendipita indica* to enhance Pi nutrition (Hiruma et al., 2016; Nizam et al., 2019; Frerigmann et al., 2021). Colonization by *C. tofieldiae* is controlled by the PSR response and the host’s production of tryptophan-derived indole glucosinolates (IG) as defence compounds keeps the extent of colonization in check (Hiruma et al., 2016; Frerigmann et al., 2021). Inability to synthesize IG (through loss of cyp79b2 cyp79b3 function) enables *C. tofieldiae* to behave as a pathogen (Hiruma et al., 2016). Pi-starvation can lower levels of IG in roots (but not shoots) consistent simplistically with the model of a lowering of plant defences (Frerigmann et al., 2021). Colonization increases levels of 4-methoxy-indole-3-methyl-glucosinolate which would require the activity of the P450 monooxygenases CYP83B1, CYP81F2, CYP81F3 and the Indole Glucosinolate O-Methyltransferase IGMT2 (Frerigmann et al., 2021). Recently eATP has been found to act through P2K1 (albeit in Pi-replete seedlings) to upregulate expression of the genes encoding those key enzymes (Jewell et al., 2022). This leads to the speculation that damage incurred by colonization could signal to effect the IG response, with the further possibility that ATP secreted by the fungus in this and other invasive scenarios could contribute to signalling. A first step would be to see if eATP modulates IG synthesis under Pi deprivation. The early phase of *S. indica* infection (albeit in Pi-replete roots) increases eATP and P2K1 helps limit colonization (Nizam et al., 2019). Over time the fungus secretes an eATP hydrolysing ecto-5´-nucleotidase (E5´NT) that can reduce eATP levels and promotes colonization, indicating that eATP signalling may ultimately need to be impaired (Nizam et al., 2019). Indeed, expressing the *S. indica* nucleotidase in *Arabidopsis* roots rendered them more susceptible to colonization by the pathogenic fungus *C. incanum* (Nizam et al., 2019). Modelling of Pi and sugar fluxes between *Arabidopsis* and *S. indica* suggests that if host ATP release were low, the fungal E5´NT could contribute to host Pi nutrition with no Pi cost to the fungus, only sucrose benefit. With high ATP release, E5´NT could contribute to Pi uptake of both host and fungus (Nizam et al., 2019). It seems in the fungus’ survival benefit to hydrolyze the eATP signal but as P2K1 is a high affinity receptor (dissociation constant 46 nM; Choi et al., 2014), eATP levels would have to be negligible to avoid triggering the pathway and there is evidence that P2K1 could still operate in [Ca^2+^]_cyt_ signalling of Pi-starved roots (Matthus et al., 2022). Perhaps it is the loss of the P2K1-independent [Ca^2+^]_cyt_ signal in Pi-starved roots that is critical to dampening the eATP defence pathway. The fungal endophyte could also modulate the eATP pathway and it is notable that although P2K1 acts to limit *S. indica* colonization (Nizam et al., 2019) this fungus does not cause the peroxide accumulation typical of eATP signalling (Camehl et al., 2011). Moreover, its cell wall extracts suppress expression of CNGC2 (Vadassery et al., 2009), a key component of eATP signalling (Wang et al., 2022a). Eat the signal and perturb the pathway.

## Conclusions and prospects

Even under Pi-replete conditions plants must regulate eATP to render it an effective signal and avoid cell death. Under Pi deprivation, the balancing act may have to include the diminution of the endogenous cytosolic ATP supply and the salvage of eATP to bolster Pi nutrition. That eATP can still trigger a modified P2K1-dependent [Ca^2+^]_cyt_ response in Pi- starved roots argues for a robust signalling system that is modulated to allow perhaps for beneficial colonisation. If “net” eATP were lower under Pi deprivation, even after wounding, then much depends on the receptors involved and their affinities, the eATP that could be produced by microbes and the ability of the microbes to degrade host or their own eATP. Whilst *Arabidopsis* remains the most well defined and tractable system, there is a clear need to resolve eATP signalling systems in crops and the impact of Pi deprivation.

## Data availability statement

The original contributions presented in the study are included in the article. Further inquiries can be directed to the corresponding author.

## Author contributions

All authors listed have made a substantial, direct, and intellectual contribution to the work and approved it for publication.
